# The development of live biotherapeutics against *Clostridioides difficile* infection towards reconstituting gut microbiota

**DOI:** 10.1080/19490976.2022.2052698

**Published:** 2022-03-23

**Authors:** Yongrong Zhang, Ashley Saint Fleur, Hanping Feng

**Affiliations:** Department of Microbial Pathogenesis, School of Dentistry, University of Maryland, Baltimore, MD 21201, United States

**Keywords:** *Clostridioides difficile*, microbiota, biotherapeutics, dysbiosis, reconstitution

## Abstract

*Clostridioides difficile* is the most prevalent pathogen of nosocomial diarrhea. In the United States, over 450,000 cases of *C. difficile* infection (CDI), responsible for more than 29,000 deaths, are reported annually in recent years. Because of the emergence of hypervirulent strains and strains less susceptible to vancomycin and fidaxomicin, new therapeutics other than antibiotics are urgently needed. The gut microbiome serves as one of the first-line defenses against *C. difficile* colonization. The use of antibiotics causes gut microbiota dysbiosis and shifts the status from colonization resistance to infection. Hence, novel CDI biotherapeutics capable of reconstituting normal gut microbiota have become a focus of drug development in this field.

## 1. Introduction

In 1935, Ivan C. Hall et al. isolated a new bacterial species from the feces of healthy newborn infants. The bacteria discovered in this study was named *Bacillus difficilis* due to the difficulty encountered in its isolation and culture.^1^ It was classified as *Clostridium difficile* in the 1970s, and now *Clostridioides difficile*.^1,2^ Hall et al.’s study is the first documented case of *C. difficile* in human intestinal microbes. Later, more studies reported the isolation of *C. difficile* from healthy infants and asymptomatic adults which endowed the bacterium with the role of normal intestinal commensal. In fact, *C. difficile* colonization is found in up to 15% of healthy adults, and its prevalence is even higher in hospitalized patients and residents of long-term care facilities.^3–5^ In the 1970s, as the surge of antibiotic-associated colitis increased, toxin-producing *C. difficile* was eventually identified as one of the major pathogens responsible for the disease.^6^ Two exotoxins, toxin A and B (TcdA and TcdB), are its main virulence factors that can disrupt the architecture of the intestine and induce severe inflammation.^7,8^ *C. difficile* has been listed as one of the top antibiotic resistance threats to public health by the Center for Disease Control and Prevention (CDC).^9^ The clinical spectrum of CDI can vary widely, ranging from asymptomatic colonization of the gastrointestinal (GI) tract to severe disease leading to toxic megacolon or intestinal perforation. Based on CDC guidelines, asymptomatic colonization does not require any treatment. Symptomatic CDI often occurs after antibiotic treatment and long-term hospitalization. It is believed that the perturbation of homeostasis of gut microbes by antibiotic treatment is highly correlated to the infection.

The human GI tract is naturally a huge reservoir of microbes in which trillions of microorganisms inhabit. This group of microorganisms is called gut microbiota (Box 1). The interaction between microbiome (Box 1) and host is absolutely a hotspot of research in recent years. It has aroused extensive attention and been widely studied not only in infectious diseases but also in other disorders.^10,11^ As technological advances and in-depth understanding of the gut microbiome have increased in the past decade, research has revealed that host-residential bacteria interaction may influence the formation of host neurological systems, nutrition metabolization, and host immune responses.^9–12^ In addition, recent clinical studies demonstrated that the gut microbiota may affect the efficacy of oncology medications in patients.^13,14,15^ Although under normal circumstances the host and gut microbiome are mutually beneficial, dysbiosis (Table 1) of the gut microbiota has been associated with many diseases.^10–12^ Recently, significant progress has been made in developing live biotherapeutics that help to restore the normal gut microbiota and treat CDI. In this review, we briefly summarize the role of gut microbiota in the pathogenesis of CDI and discuss the current development of live biotherapeutics against CDI.

**Table 1.** Glossary

TERMS	DEFINITION
MICROBIOTA	Microorganisms, composed of bacteria, fungi, virus, protozoa and archaea, inhabiting a defined environment.
MICROBIOME	Generally, microbiota, its genes, gene p roducts and activities in niches in a habitat.
METABOLOME	A large array of small molecule metabolites produced by microbiota into the inhabited environment during the metabolism of food and xenobiotics. In the case of the gut metabolome, metabolites from both microbiota and hosts should be included.
DYSBIOSIS	There is no consensus that defines dysbiosis despite a high frequency of usage in microbiome studies. It is often described as a state, in which alterations to the microbiota of hosts and its functional components may be correlated with undermined host immunity and increasing susceptibility to diseases, for example blooming of *C. difficile*. Dysbiosis usually features: i) impaired microbial diversity; ii) loss of beneficial commensal bacteria; iii) thriving of pathogens.

## 2. Gut microbiota and *C. difficile* infection

**Figure 1.**
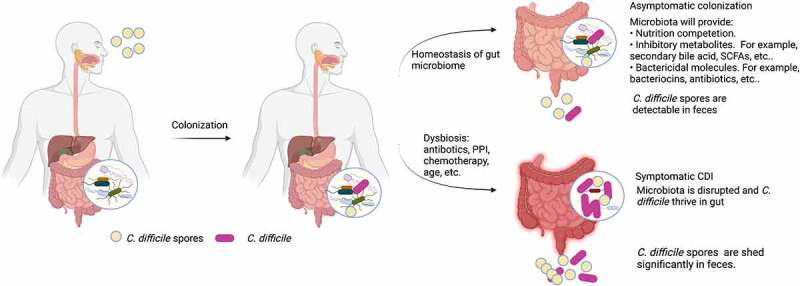
The role of gut microbiota in CDI development.

*C. difficile* survives in harsh environments in the form of spores that are highly tolerant to heat, oxygen, ultraviolet light, common disinfectants, and antibiotics.^16^ Ubiquitous spores in the environment, especially in hospitals and healthcare facilities, are the leading source of *C. difficile* transmission via the oral-fecal route. Healthy individuals upon oral ingestion of *C. difficile* spores may not develop any sign of disease but shed spores and bacterial debris in their feces, which was defined as colonization by Crobach *et al*.^17^ Colonization does not necessarily proceed to symptomatic infection. In fact, an intrinsic homeostasis in the gut microbial niche allows a resistance to *C. difficile* colonization and infection (Figure 1). Within the niche, the microorganisms benefit each other but competitively restrain the colonization of opportunistic pathogens at the same time. ‘Good’ bacteria build up an ‘unfavorable’ environment in which *C. difficile* are not able to expand and thrive. Although persistent colonization as asymptomatic carriage can last for months, as long as the homeostasis of the gut microbiome is maintained, infection may not occur.^5,18^ Based on the current knowledge, gut resident commensals contribute to homeostasis conferring resistance to symptomatic infection in three major mechanisms including nutrition competition, production of inhibitory metabolites and secretion of bactericidal molecules.^18–22^ These mechanisms have been well discussed in other reviews^23,24,25^ and therefore will not be discussed in this review.

CDI often occurs upon disruption of the homeostasis of gut microbiota (Figure 1). Loss of the total number of gut microorganisms and diversity of microbiota have been frequently reported in CDI patients.^26,27^ Perturbation of gut microbiota will consequently lead to an imbalanced living environment, including altered metabolome, pH, and epithelial mucus, of which opportunistic pathogens like *C. difficile* will take advantage to expand.^27–29^ Once *C. difficile* dominates, it will produce two exotoxins that will disrupt the intestinal epithelium and cause inflammation.^7,8^ Meta-analysis showed that carriers of toxigenic strains are at a higher risk for the development of an infection compared to non-colonized patients.^26^ Therefore, colonization with toxigenic *C. difficile* and dysbiosis are prerequisites of CDI. Direct perturbation of microbiota is mainly induced by antibiotics and proton pump inhibitors (PPIs), while some chemotherapies and advanced age may also affect the intestinal microbiome components.^31–33^ Among them, antibiotic treatment is still the leading risk of primary and recurrent CDI.

Since gut dysbiosis leads to the loss of *C. difficile* colonization resistance, therapeutic strategies aiming to restore a normal gut microbiota have gained attention in the past decade and achieved some remarkable success. These strategies are pleiotropic as they not only restore the microbiome structure but also recover its biofunctions. When the gut residential microbe community recovers, the biofunctions that produce a normal metabolome will be re-established and shift to *C. difficile* colonization resistance as described earlier in this review. To date, these strategies mainly include full fecal microbiota transplantation (FMT) and precise fecal bacterial/probiotic strain(s) engraftment. Strategies directly targeting *C. difficile* are also emerging. In addition, compounds, such as ribaxamase and prebiotics, have been investigated for their ability to manipulate the gut microbes to defend against CDI.^34–36^

In next sections, we will summarize the interventions using live microorganisms to treat CDI that directly or indirectly restore the homeostasis of the gut microbiome, including their mechanisms, preclinical and clinical progress, and benefits versus risks.

## 3. FMT and its derivatives

FMT treatment for diarrhea can trace its history back to 1700 years ago by a Chinese physician.^37^ The first FMT used to treat CDI-related pseudomembranous colitis was recorded in 1958.^38^ Not until the past decade, have FMT and its derivatives been extensively studied and widely applied in patients with CDI, particularly those with recurrent CDI. Numerous pre-clinical and clinical studies have demonstrated the promising efficacy of FMT and its derivatives in treating CDI, although differences exist between the regimens (Table 2).

**Table 2.** Key features of FMT and its derivatives

	SOURCE	CULTIVATED	COMPOSED STRAINS	DOSAGE OF EACH COMPOSITION
TRADITIONAL FMT	HD^a^	No	Unknown	Unknown
DEFINED FMT	HD/CP^b^	Yes	Designated bacteria mixture	Designated
FMT SPORES	HD	No	*Firmicutes* species	Unknown

a: healthy donors; b: FMT cured CDI patients.

### 3.1 FMT

Utilizing a dynamic computer-controlled in vitro model of the human colon, a very recent study has dissected how FMT rescues the gut microbiome from antibiotic therapy-induced loss of microbial diversity and abnormal bioactivities.^30^ Shortly after FMT application, the fermentation activity, measured by pH and redox potential, gas production and short-chain fatty acids (SCFA) production, quickly recovered to baseline. It also shortened the recovery time of the bacterial profile at both diversity and richness levels.^30^ Similar to *C. difficile* treatment, restoration of normal colonic microbial ecology by FMT restores bile acid metabolism and normal bile acid composition in the colon, producing an unfavorable environment for *C. difficile* spore germination and allowing clinical recovery of recurrent CDI patients.^39,40^ Jillian R.-M. Brow et al. further found that the ratio of inflammatory to non-inflammatory fatty acids decreased although the total fatty acid levels were restored in patients’ gut.^40^ Patients post-FMT developed similar microbial structure as donors’ for a certain period.^41^ Another clinical study also showed that FMT dramatically reduced the abundance of antibiotic-resistant bacteria in the 2 months after administration, suggesting fecal antibiotic resistance gene carriage decreased in direct relationship to the degree to which donor microbiota was engrafted.^42^

The above findings and many more have established FMT as an evidence-supported treatment option for recurrent CDI. According to the 2017 IDSA/SHEA clinical practice guidelines, FMT is recommended for patients with multiple recurrences of CDI.^43^ The development of FMT therapeutics is riding on the crest of a wave. Most of the registered clinical trials in the United States targeting gut microbiome to treat CDI or recurrent CDI are FMT (Table 3). Several whole-stool FMT products in the pipeline including RBX2660, VE303, CP101, and RBX7455 have been in clinical trials. Among them, RBX2660 is the only one now in clinical trial phase 3. As a commercial FMT regimen, RBX2660 showed favorable clinical efficacy and safety results in its phase 2 clinical trials.^44,45^

**Table 3.** Registered clinical trials use live microorganisms as interventions to treat CDI in the United States

NCT Number	Status	Conditions	Intervention	Commercial name	Phase	Ref.
NCT04090346	Enrolling by invitation	Recurrent CDI	FMT		Phase 4	
NCT03973697	Recruiting	Recurrent CDI	FMT		Phase 2	
NCT03970200	Recruiting	Severe CDI	FMT		Phase 2	
NCT03931941	Recruiting	Recurrent CDI	FMT	RBX2660	Phase 3	
NCT03829475	Recruiting	IBD/CDI	Bezlotoxumab and/or FMT		Phase 2	
NCT03795233	Suspended	CDI	FMT		Phase 1&2	
NCT03788434	Recruiting	Recurrent CDI	FMT	VE303	Phase 2	
NCT03621657	Completed (has results)	CDI	FMT		Phase 2	
NCT03617445	Suspended	Recurrent CDI	FMT		Phase 2	
NCT03548051	Terminated	CDI	FMT		Phase 1&2	
NCT03497806	Active, not recruiting	CDI and recurrent CDI	FMT	CP101	Phase 2	
NCT03298048	Terminated Has Results	Recurrent CDI	FMT		Phase 2	
NCT03268213	Active, not recruiting	CDI/UC/indeterminate colitis	FMT		Early Phase 1	
NCT03244644	Completed	Recurrent CDI	FMT	RBX2660	Phase 3	
NCT03183141	Recruiting	Recurrent CDI	FBS^a^	SER-109	Phase 3	
NCT03183128	Completed	Recurrent CDI	FBS	SER-109	Phase 3	^131^
NCT03110133	Completed	Recurrent CDI	FMT	CP101	Phase 2	
NCT03106844	Completed	IBD/CDI	FMT		Phase 1&2	
NCT03005379	Recruiting	Recurrent CDI	FMT		Phase 2&3	
NCT02981316	Completed	Recurrent CDI	FMT	RBX7455	Phase 1	^132^
NCT02589964	Terminated	CDI	Probiotic	Florajen-3	Phase 1	
NCT02589847	Completed	Recurrent CDI	FMT	RBX2660	Phase 2	
NCT02465463	Completed	CDI	FMT		Phase 1&2	
NCT02437487	Completed	Recurrent CDI	FBS	SER-109	Phase 2	^56^
NCT02423967	Completed	Recurrent CDI	FMT		Phase 1	
NCT02403622	Terminated	Recurrent CDI	FMT		Phase 2	
NCT02343328	Terminated	Recurrent CDI	FMT		Phase 1	
NCT02299570	Completed	Recurrent CDI	FMT	RBX2660	Phase 2	^44,45^
NCT02269150	Active, not recruiting	Prophylaxis of CDI	FMT		Phase 2	^133^
NCT02255305	Recruiting	Recurrent CDI	FMT		Phase 2	
NCT02134392	Recruiting	Recurrent CDI	FMT		Phase 1	
NCT02127398	Recruiting	Recurrent CDI	FMT		Phase 2	
NCT01972334	Completed	Recurrent CDI	FMT		Phase 2	
NCT01925417	Completed	Recurrent CDI	FMT	RBX2660	Phase 2	^42,134^
NCT01914731	Completed	Recurrent CDI	FMT		Phase 1	^135^
NCT01868373	Enrolling by invitation	Primary and recurrent CDI	Defined FMT		Phase 1	
NCT01704937	Completed	Recurrent CDI	FMT		Phase 1	^136^
NCT01680874	Completed	Primary and recurrent CDI	Probiotic^b^		Phase 2	^137,138^
NCT01259726	Completed	Prevention of recurrent CDI	NTCD	VP20621	Phase 2	^92^
NCT01202630	Suspended	Recurrent CDI	Probiotic^c^	Bio-K, CL1285	phase 3	
NCT02127814	Completed	AAD, CDI	Probiotic^d^		NA^a^	
NCT03562741	Recruiting	Recurrent CDI	FMT		NA	
NCT02830542	Unknown	Recurrent CDI	FBS	SER-262	Phase 1	
NCT02636517	Active, not recruiting	CDI, IBD, UC, Crohn’s Disease	FMT		NA	
NCT02557685	Unknown	CDI	FMT		Phase 2	
NCT02326636	Completed	CDI	FMT		NA	
NCT02076438	Terminated	AAD, CDI	Probiotic^e^	Culturelle	NA	
NCT01973465	Unknown	CDI	FMT		NA	
NCT01905709	Recruiting	CDI	FMT		NA	
NCT01873872	Unknown	CDI	Probiotics	Theralac & Culturelle	NA	
NCT01703494	Unknown	CDI	FMT		Phase 2	^41^
NCT01077245	Withdrawn	CDI	Probiotic CBM588	MIYA-BM	Phase 2	

a: FBS = Fecal bacterial spores; NA = Not applicable. b: equal amounts of Lactobacillus acidophilus NCFM® (ATCC 700396), Lactobacillus paracasei Lpc-37 (ATCC SD5275), Bifidobacterium lactis Bi-07 (ATCC SC5220), and Bifidobacterium lactis Bl-04 (ATCC SD5219). c: Lactobacillus acidophilus CL1285® and Lactobacillus casei. d: Lactobacillus reuteri. e: Lactobacillus Rhamnosus GG

Although highly efficacious, FMT is associated with risks, such as heterogeneity among donors, batches and preparations, and unknown long-term impact post FMT treatment.^46^ The major risks of FMT include: 1) Side effects, such as constipation, diarrhea, bloating, etc., often occurred after transplantation; 2) It may transfer potential pathogens. Although the donors have been screened for recognized pathogens, uncertain pathogens may still exist. The screening procedure for donors was updated by the United States Food and Drug Administration (FDA) in 2019 because of cases of invasive infection caused by extended-spectrum beta-lactamase (ESBL)-producing *Escherichia coli* in two immunocompromised patients after FMT;^47^ 3) Disorders or diseases other than infectious disease may be transferred to the recipients. Most of the clinical trial studies only have a 6-month follow-up period, and no data so far have demonstrated the long-term impact of FMT. As we discussed in the beginning, based on current knowledge, the gut microbiome plays a vital role in many aspects of human physiological functions. Therefore, it is hard to predict whether the donors may transfer their own characteristics to the recipients, for instance, obesity, diabetes, colon carcinoma, neurological disorders, etc.

### 3.2 Defined FMT

The defined FMT is intrinsically a precise engraftment with a designated fecal bacterial composition. As an alternative to FMT, the defined FMT has multiple advantages. First, the bacterial composition is designable and controllable. The absence and presence of certain bacterial strains are frequently and consistently observed in CDI patients pre- and post-therapy. Thus, replenishment of those bacteria that defined a healthy state may inhibit CDI *in vivo*. Tvede and Rask-Madsen initiated the proof-of-concept study in which they treated 5 CDI patients through rectal administration with a mixture of equal volume of 10 bacterial cultures isolated from the feces of healthy donors.^48^ The strains were chosen based on their characteristics as probiotics, their inhibitory effects to C. difficile *in vitro*, and absence in CDI patients. Similarly, Cammarota et al. introduced a 15-strain consortium derived from successful engraftments in patients cured by FMT.^49^ For both studies, in response to the treatment procedure, patients were promptly relieved of CDI symptoms. Second, the defined FMT can lower the risk of antibiotic resistance. Petrof and colleagues adopted a regimen possessing a broader spectrum of intestinal microbes (33 strains).^50^ Unlike Tvede’s and Cammarota’s, the strains in Petrof’s study were prepared to mimic fecal microbiota. The selected strains (except Bifidobacterium spp.) were formulated in an inferred relative ratio based on the MetaREP metagenomic database of stool sample datasets from healthy donors. The authors also considered antibiotic susceptibility and safety, while attempting to generate as much taxonomic diversity as possible. If any selected organism was suspected to be resistant to antibiotics, then it would be ruled out of the mixture. Third, the regimen is reproducible, low cost, stable and can be quality controlled. Variations of the FMT products widely exist between individual donors. Even the same donor may not produce identical microbiota at different times. Therefore, reproducibility is one of the biggest pitfalls for FMT products. However, the defined FMT is a synthetic bacterial mixture cultured in vitro that can be easily produced and genetically monitored. For example, in Petrof’s study, all purified candidates were identified by 16S rRNA sequencing to guarantee the genomic background. Since all bacteria are culturable in vitro, the time and cost required for donor screening will be saved. Fourth, an absence of viruses and other pathogens in the administered mixture can be ensured, thereby improving patient safety.^50^

Despite these advantages, challenges also exist. In the United States, only one registered ongoing clinical trial using defined FMT as intervention was found on the Clinical trials website (Table 1). In Europe, the result of a randomized controlled clinical trial (RCT) was released recently. In this study, a total of 98 participants with recurrent CDI were enrolled, which presents the largest trial on this topic to date. The treatment did not show a superior efficacy compared to conventional FMT (cure rate 52% vs 76%), although it was comparable to vancomycin treatment (cure rate 52% vs 45%).^51^ The regimen of 12 well-characterized bacterial strains, selected only based on previous experiences, was a simple equal volume mixture of bacterial candidates. Nowadays, as the development of metagenomic and culturomic techniques progresses, more bacterial strains have been isolated from gut microbiota and identified to be closely related to CDI dysbiosis.^52,53^ Species composition and interactions are both important determinants of the C. difficile inhibition phenotype, as a simple cocktail of selected strains may not achieve ideal inhibitory outcomes but oppositely may assist C. difficile growth.^53^ Therefore, regarding the interaction of bacteria with each other in the mix and with other members of gut commensals of hosts, a personalized bacterial cocktail may be required to treat individuals.

### 3.3 Fecal bacterial spores

Another FMT derived strategy is the mixture of fecal bacterial spores. SER-109, a pipeline product of Seres Therapeutics, Inc., is approaching the market. The company has released inspiring primary endpoint results from its phase 3 trial using SER-109 to treat recurrent CDI. The patients had a significantly lower recurrence rate of 11.1% in the SER-109 treatment group versus 41.3% in the placebo group 8 weeks after treatment.^54^ The drug is a consortium of spores of multiple *Firmicutes* species fractionated from the stools of healthy human donors and encapsulated.^55,56^ *Firmicutes* play an important role in colonization resistance.^57^ The SER-109 was manufactured with a sterilization process to reduce potential pathogens and fecal matter and debris. The entire spore ecology of SER-109 is more physiologic than defined FMT as all healthy donors’ spores were represented without depleting or enriching for specific spore species based on sequencing and microbiology studies. However, SER-109 had failed to achieve its primary endpoint in phase 2 in 2016. The chief scientist of the company revealed two important modifications for their phase 3 study: detection method for *C. difficile* and dosage of SER-109. Instead of amplifying *C. difficile* genes, ELISA for detecting toxins was performed to differentiate active infection and asymptomatic colonization, implying that the microbiota niche shifted back to a balanced state of colonization resistance after treatment.^58^ The dosage of the drug was also emphasized to play a critical role in its efficacy.^58^

## 4. Probiotics

The current consensus definition of probiotics is “live strains of strictly selected microorganisms that, when administered in adequate amounts, confer a health benefit on the host” by an expert panel of the International Scientific Association for Probiotics and Prebiotics (ISAPP) since October 2013.^59^ According to the consensus, probiotics should be well-known strains that provide health benefit in single or multiple mechanisms. Hence, the defined FMTs described in a previous section of this review indeed meet the criteria of a probiotic.^59^ Since the initial aim of defined FMTs was to mimic fecal microbiota, we categorized them as FMT derivatives. In fact, as the in-depth study of FMT advances, the gut commensal will be a source of next-generation probiotics.^60^ The wide range of the probiotic definition will surely encourage innovation in the field. Although not all mechanisms of probiotics have been confirmed in humans, diverse mechanisms are likely to drive probiotic benefits to host health, such as production of antimicrobial products, cross-feeding the resident commensal, direct interaction with immune cells, etc.^61^ In this section, we will summarize the non-FMT probiotic-related interventions in CDI prevention.

### 4.1 Traditional probiotics

The traditional probiotics, such as strains of *Lactobacillus, Bifidobacterium*, and *Saccharomyces* have been suggested as dietary supplements for CDI.^62^ The mechanisms of probiotics against CDI are similar to FMT. A consortium of probiotics, including five *Lactobacilli* strains, two *Bifidobacterium* standard strains, and *Bifidobacterium infantis* obstructs the proliferation of *C. difficile* through affecting the diversity of gut microbiota and regulating SCFA production, eventually attenuating *C. difficile* colonization.^63^ *Lactobacillus* and *Bifidobacterium* species have also been shown to colonize the intestine regardless of concurrent antibiotic use, competing with *C. difficile* for nutrition.^64,65^ *Saccharomyces boulardii* was reported to lessen antibiotic induced microbiota shifts.^66,67,68^ In addition, *S. boulardii* produces a protease capable of digesting *C. difficile* toxins, which are etiologies of the disease, and modulates a host of inflammatory signaling pathways to inhibit toxin-induced inflammation.^76–79^

The traditional probiotics have a long history as safe and effective diet supplements or drugs with health benefit. A major outbreak, associated with the *C. difficile* NAP1/027/BI strain in the hospital Pierre-Le Gardeur (PLGH) in Quebec, promoted the use of probiotic Bio-K+, containing three *Lactobacillus* strains, to every adult inpatient on antibiotics in this hospital. The data collected by the Ministry of Health in Quebec showed no episodes of *Lactobacillus* bacteremia during the entire 10-year experience and that CDI incidents were lower in this hospital.^70,71^ Although in particular immunocompromised individual treatment with *S. boulardii* should be avoided or well managed due to fungemia concerns,^72,73^ it has been used to prevent and treat diarrheal diseases such as antibiotics associated with diarrhea (AAD) for many decades.^74,75^ Hence, the overall safety of using traditional probiotics to treat CDI is not a major concern. Moreover, the probiotic products can be easily and economically prepared and given to patients daily as yogurt, drinks, cheese, or capsules.^64,85–87,88,89^ Unlike FMT bacteria, the probiotics are frequently used as prophylactics or adjuvant therapy to reduce the risk of AAD and/or primary CDI in many clinical trials across the world. Inconsistent conclusions drawn from these clinical trials may be the reason that probiotics are not recommended for CDI treatment and prevention by current clinical practice guidelines. However, a meta regression analysis emphasized the efficacy of probiotics in reducing CDI incidence in a high-risk population.^79^ In this study, a total of 19 published RCTs, comprising 6261 subjects and using different regimens of four probiotic species (*Lactobacillus, Saccharomyces, Bifidobacterium*, and *Streptococcus*), were analyzed. The authors found that the timing of probiotic administration was critical to prevent CDI. Future research is still needed to focus on optimal probiotic dose, species, and formulation since no superior regimens were concluded in this study.

### 4.2 Newly emerging commensal probiotics

Given the broad definition of probiotics, several commensal bacteria may now be categorized as probiotics since they have been frequently demonstrated to play important roles in combatting CDI. Most of them are in the preclinical stage of development. In order for the emerging probiotics to assert benefits in CDI patients, more clinical studies are needed to examine criteria, such as dose responses, biological plausibility, replication of findings, etc.^59^ Here are some examples of the newly identified probiotic candidates.

*Clostridium scindens* was identified as a probiotic candidate from commensals to treat CDI through a subtle workflow described by Buffie and colleagues.^80^ They first analyzed the microbiota composition in mice after different antibiotic regimens and identified 11 strains that were strongly associated with *C. difficile* colonization resistance. Then, an inference-modeling approach was applied to samples of hospitalized patients in parallel with the murine samples. *C. scindens* was identified as the strongest correlated bacteria to enhance resistance to CDI in both human and murine CDI. Further study indicated that *C. scindens* mediated inhibition was bile acid dependent.

CBM588 is a probiotic consisting of *Clostridium butyricum*, a bacterium that produces a robust amount of butyrate. It has been used as a live biotherapeutic probiotic to treat CDI patients in a phase 2 clinical trial, although the trial was eventually suspended due to lack of enrollment. Preclinical studies showed that *C. butyricum* activated neutrophils and Th1 and Th17 cells to elicit the protective effects against CDI.^81^

Li et al. investigated the role of *Bacteroides thetaiotaomicron* in defending against CDI in a mouse model. Similar to the FMT control, mice administered *B. thetaiotaomicron* had fewer copies of *C. difficile* as well as less colonic inflammation. Meanwhile, both FMT and *B. thetaiotaomicron* improved the gut microbiota composition and reversed the CDI-induced change in bile acid composition, suggesting that *B. thetaiotaomicron* is a good probiotic candidate to combat CDI.^82^ The cell-wall associated glycans of *B. thetaiotaomicron* were shown to suppress the production of the glycosylated toxins of *C. difficile* in vitro.^83^ As a cephalosporinase-producing anaerobe, the pre-colonized *B. thetaiotaomicron* produced β-lactamase enzymes to inactivate intraintestinal β-Lactam antibiotics during antibiotic treatment, providing protection for commensal recovery and preventing overgrowth of *C. difficile* in a mouse model.^84^

## 5. Non-toxigenic *C. difficile* spores

Nontoxigenic *C. difficile* (NTCD) strains that lack the genes for active toxin production are frequently found in the hospital environment and colonize hospitalized patients, although patients are usually asymptomatic for CDI. One hospital study found that asymptomatic colonized patients had less chance of developing active CDI,^85^ implying pre-colonization with NTCD would be a potential treatment to protect hospitalized patients from recurrent CDI. Several preclinical studies have demonstrated the efficacy of NTCD or low-virulent *C. difficile* to prevent CDI.^95–99^ The mechanism might be that a certain population of NTCD occupies the living space and outcompetes the invading strains. Recently, Lesile et al. revealed that consumption of glycine by the first colonized strain of *C. difficile* would decrease germination of the second lethal strain, consequently limiting colonization by the lethal one.^90^

VP20621, a commercial product of Viropharma Inc, is an oral liquid drug containing the spores of NTCD strain M3 and has completed phase 2 clinical trial. The released results indicated that the drug was quite safe and tolerable as patients took daily dosages ranging from 10^4^ to 10^8^ CFU for 7 or 14 days and only had similar mild side effects.^91,92^ NTCD-M3 was isolated from human patients.^87^ In its phase 2 trial, the average fecal colonization rate of NTCD-M3 was 69% (71% with 10^7^ spores/d and 63% with 10^4^ spores/d). Recurrence of CDI occurred in 13 (30%) of 43 placebo patients and in 14 (11%) of 125 NTCD-M3 patients. The NTCD-M3 colonization became completely undetectable after week 22 of follow-up, implying the restoration of the normal microbiota, which may then provide protection against subsequent CDI. Notably, the dosage of 10^7^ spores/d for 7 days had a lower recurrence than the dosage of 10^7^ spores/d for 14 days. The gut microbiome profile alteration between pre- and post-spore treatment needs further investigation to dissect how NTCD competes against invasive *C. difficile* and restores the microbiota.

At the time of writing, NTCD-M3 is the only biotherapeutic using single-species bacteria that has demonstrated efficacy in reducing recurrent CDI in the clinic. Compared to FMT-related strategies, the NTCD-M3 has a clearer genetic background. Preparation is reproducible and low cost. However, there are a couple of concerns about widespread clinical use, such as antibiotic resistance gene transfer and toxin gene acquisition.^93^ Horizontal gene transfer occurs both between bacteria of the same species and between different species through variant mechanisms.^94–96,^*C. difficile* strains are known to be resistant to a wide spectrum of antibiotics. Extensive use of a highly antibiotic resistant strain of NTCD would increase the risk of spreading antibiotic resistance to other bacteria in the gastrointestinal tract. Meanwhile, the co-colonized toxigenic *C. difficile* strain may convert the NTCD to a toxin producer strain by horizontal gene transfer.^97^

## 6. Bacteriophages

Bacteriophages are viruses that can infect and replicate within their host bacteria and eventually lyse their hosts. Although the use of *C. difficile*-specific bacteriophages does not directly target gut microbiota, this approach precisely targets *C. difficile* without using antibiotics. To date, no treatment against CDI using bacteriophages is in clinical practice, although they have been widely used in humans in European countries to treat other infections.^98^ Several preclinical studies have demonstrated bacteriophages and phage-derived products as potential therapeutics to inhibit *C. difficile* growth and toxin production both in vivo and in vitro.^99–102^ Although these results are promising, there are several drawbacks with the current bacteriophage strategies. All the *C. difficile* phages with complete genomes in the public database are temperate phages, which can be replicated either by the lytic or the lysogenic cycle.^103^ When the temperate phage goes into a lysogenic cycle, it becomes integrated into the host genome to replicate with the host chromosome or replicate within the host as a plasmid.^103^ The lysogenic cycle creates phage resistance, leaving the host *C. difficile* strain tolerant to the phage mediated eradication. Genetically engineered phages that are deficient of lysogen-related genes seemed to be a solution. Selle et al. developed an engineered *C. difficile* phage carrying a self-targeting CRISPR-Cas array to directly target the bacterial chromosome.^104^ This recombinant phage was demonstrated to be more efficient at killing *C. difficile* both *in vitro* and *in vivo* while lysogens still accumulated 24 hours post infection. To avoid lysogeny formation, the authors removed a region of the genome encoding the cI repressor and integrase gene (wtPhage Δlys and crPhage Δly). Although no lysogeny from in vitro infection with wtPhage Δlys and crPhage Δly was detected, lysogens appeared in feces of mice treated with each, suggesting other unknown mechanisms may exist to drive the lysogenic replication cycle in *C. difficile* phages. Unfortunately, this particular characteristic of phages may play an important role in horizontal gene transfer that contributes to bacterial evolution and may generate superbugs. Phages transferring antibiotic resistance to their host strains have been demonstrated. Goh et al. found that phage phiC2 was able to promote the transfer of the transposon Tn6215, which encodes erythromycin resistance, between *C. difficile* strains.^105,106^ Furthermore, *C. difficile* toxins may also originate from phages. The complete functional binary toxin locus was identified in the genome of phage phiSemix9P1.^106^ Besides, use of phage may risk promoting biofilm since LuxS encoding enzyme mediated induction of prophages likely contributes to *C. difficile* biofilm structure.^107^ Finally, the host specificity as its advantage is also a disadvantage for phage therapy. There is not a universal phage regimen that can kill all *C. difficile* variants. Hence, a personalized bacteriophage regimen may be necessary for CDI treatment.^108^ *C. difficile* isolation and sequencing may be needed prior to treatment administration to determine which phage or phage combination may be applicable to the specific patient.

Overall, bacteriophage therapy is a potential candidate to treat CDI that avoids antibiotic usage and disturbs the gut microbiome. More knowledge about the phage-*C. difficile* interaction is needed to safely translate this therapeutic concept to human patients.

## 7. Recombinant live biotherapeutic products

According to the FDA-updated guidance in 2016, recombinant live biotherapeutic products (LBPs) are composed of genetically modified microorganisms with the purposeful addition, deletion, or modification of genetic material.^109^ The genetic modifications can empower additional functions of LBPs besides their intrinsic effects on gut microbiota, and are therefore potentially more promising in fighting against CDI. Although to date, no recombinant LBPs have been approved by the FDA for human diseases, many are in development to target a range of diseases including metabolic disorders, inflammatory bowel disease (IBD), colorectal cancer, and infectious diseases.

### 7.1 Using probiotic bacteria as chassis

Probiotic bacteria such as *E. coli* Nissle 1917, *Lactobacillus* spp., *Salmonella Typhi*, etc., are often used as the chassis of the recombinant LBPs since genetic toolboxes in those bacteria have been well established.^110,111^ For example, Synlogic has developed a series of recombinant LBPs using *E. coli* Nissle 1917 as chassis to treat metabolic disorders.^112^ Another in the pipeline product of Precigen Actobio in the clinical trial phase 1b/2 for type-1 diabetes adopted *Lactococcus lactis* as chassis.^113^ In the context of CDI, two lactic acid bacterial strains, *Lactobacillus casei* and *Lactobacillus acidophilus*, engineered to display *C. difficile* surface layer protein A (SlpA) were proposed by Vedantam *et al*. to compete with *C. difficile* for epithelial adherence.^114^ The proposed strains were demonstrated to protect hamsters and piglets from death or diarrhea post infection of *C. difficile* strain 630. SlpA is the most abundant component of *C. diffic*ile cell wall and mediates the attachment of the bacteria to mucus layer of host intestine that may contribute to colonization.^115^ Vaccine studies have shown SlpA as a candidate to elicit protective immune responses against CDI in mice but not hamster models.^116,117^ However, *C. difficile* has a high level of variability of SlpA between strains.^118^ Therefore, it is risky for therapeutic development to target one of the SlpA variants solely.

### 7.2 Using probiotic yeast as chassis

Probiotic yeast *S. boulardii* has not been broadly used as a live vector for the delivery of therapeutic proteins until 2020 since the first report in 2013. ^119–123^ As a probiotic, the treatment efficacy of *S. boulardii* has already been widely assessed in various gut disorders. As an LBP chassis, *S. boulardii*’s lack of sporulation and stable colonization in the gut may further ease safety concerns.^124^ The eukaryotic yeast is unlikely to transfer antibiotic resistant genes. In addition, *S. boulardii* is tolerant to the acidic environment and grows well at 37°C.^125^ Therefore, *S. boulardii* is an attractive vehicle to deliver therapeutics in the gut. Recent genomic sequencing studies uncovered the similarities between *Saccharomyces cerevisiae* and *S. boulardii* that expands the genetic toolbox to engineer *S. boulardii*, paving the way to producing heterologous proteins in *S. boulardii*.^126^ Several studies have generated auxotrophic strains of *S. boulardii* and demonstrated the expression of heterologous proteins by this probiotic yeast.^127,128^ Chen et al. recently reported a rationally designed *S. boulardii* strain (Sb-ABAB) that has superior prophylactic and therapeutic efficacy in mouse models of primary and recurrent CDI.^122^ The concept of this design was to utilize a well-documented non-colonizing probiotic to constitutively deliver therapeutic antibodies in situ to combat intestinal colonized pathogens, such as *C. difficile*. Chen et al.’s report is the first recombinant LBP to use probiotic yeast as chassis to deliver therapeutic antibodies. *S. boulardii* may also be able to deliver other therapeutics, including bacteriocin, cytokines, peptides, and small molecular drugs.^120–123^ In order to improve the yield of target cargoes, the components of the gene cassettes, such as signal peptides, promotors/terminators, selection markers, etc., and maintenance of the exogenous gene of interests as plasmids or chromosomal integration have been modified in those studies. Among them, Chen et al. and Liu et al. have demonstrated the *in vivo* activity of the yeast-secreted heterologous proteins after passing through animal GI tract.^121,122^

Particularly to Chen and colleagues’ study, Sb-ABAB carries a plasmid harboring a nanobody-encoding gene with an uracil auxotrophic selection marker. It is also the first recombinant LBP to target *C. difficile* toxins. Several advantages exist in this immunotherapy-based recombinant LBP. First, multiple-effects-in-one confers this engineered *S. boulardii* a powerful candidate to battle *C. difficile*. Clearly, the administration of this engineered Sb-ABAB more efficiently protected the host from diarrhea and weight loss and enhanced survival compared to control vehicles. In addition to the probiotic effects described above, the delivered cargo, a tandem tetra-specific VHH antibody named ABAB, neutralized *C. difficile* toxins TcdA and TcdB simultaneously and efficiently. Although *S. boulardii* was shown to secrete an enzyme that degrades *C. difficile* toxins,^69^ ABAB is more precise and potent, targeting the toxins directly. Further analysis indicated that the secreted endogenous ABAB was stable in vivo and sufficient to decrease intestinal toxin accumulation, accompanied by a decrement of host intestinal damage and inflammation. Dramatically fewer *C. difficile* colonies were recovered from feces of mice administered Sb-ABAB, implying that colonization resistance was increased. Further exploration is needed to dissect the impact of Sb-ABAB on regulating the entire microbiome. Secondly, it is a less costly way to deliver therapeutic antibodies to protect high-risk patients from CDI. Antibody drugs are currently one of the most popular but pricy therapeutics in the market. Bezlotoxumab (commercial name Zinplava) is a human monoclonal antibody against *C. difficile* TcdB. It is the first approved antibody by the FDA for the prevention of recurrent CDI. The average wholesale price of Bezlotoxumab is $4560 per vial.^129^ The cost of antibody drugs is partially due to the complexity of manufacture. By contrast, *S. boulardii* is economical to manufacture through fermentation. Hence, utilizing the live surrogate to deliver therapeutic antibodies in a real-time fashion will reduce the manufacturing process and save costs. Thirdly, it will simplify the regimen with concurrent administration with antibiotics. Unlike other probiotic bacteria, *S. boulardii* is not suppressed by vancomycin. Therefore, when used as a prophylactic, the engineered *S. boulardii* can be taken with antibiotics in a way that will be easy to follow by high-risk patients. Finally, the clear genetic and safety background of *S. boulardii* will accelerate its translation to human patients.

There are also limitations to *S. boulardii* based recombinant LBPs. The dissemination of synthetic DNA material is a general concern of the FDA of all genetically modified LBPs, although the current Sb-ABAB adopted an auxotrophic selection marker to avoid spreading antibiotic resistance. The glycosylation profile of yeast produced proteins is different from human proteins and that may interfere with the function of therapeutics in humans, hence possibly shrinking the pool of candidates.^130^ Despite many efforts, the yield of target cargoes is still a limitation since none of the studies discussed earlier demonstrated an abundant secretion amount.

## 8. Conclusions

Due to the wide usage of antibiotics, antibiotic resistance has become a challenge in clinics to treat infectious diseases. *C. difficile* is one of the representative superbugs that is hard to treat with antibiotics. As knowledge grows, the role of gut microbiota in human health turns more transparent and gains more attention. Over the past decade, numerous studies have provided evidence to support that homeostasis of gut microbiome will shield hosts from invasion by opportunistic pathogens. Thus, ideas about reconstituting the disturbed microbiome to treat CDI are emerging. Precise and generic intestinal engraftments with various microbiota have been widely explored. Remarkable milestones have been achieved in CDI treatment using microbiota, although the long-term impact on human health is unknown. Genetically modified probiotics that specifically target *C. difficile* pathogenesis provide a brand-new direction for the treatment of this antibiotic resistant superbug. Despite a short history, microbial therapies in the *C. difficile* field open up a new era in drug development targeting gut disorders. Meanwhile, criteria about using microbiota are also in urgent need to unify the application in patients.

## Supplementary Material

Supplemental Material
