# Supplementary figures and images for: The development of live biotherapeutics against Clostridioides difficile infection towards reconstituting gut microbiota
Source: Gut Microbes. 2022 Mar 23;14(1):2052698. doi: 10.1080/19490976.2022.2052698 (PMC8959509; doi:10.1080/19490976.2022.2052698)

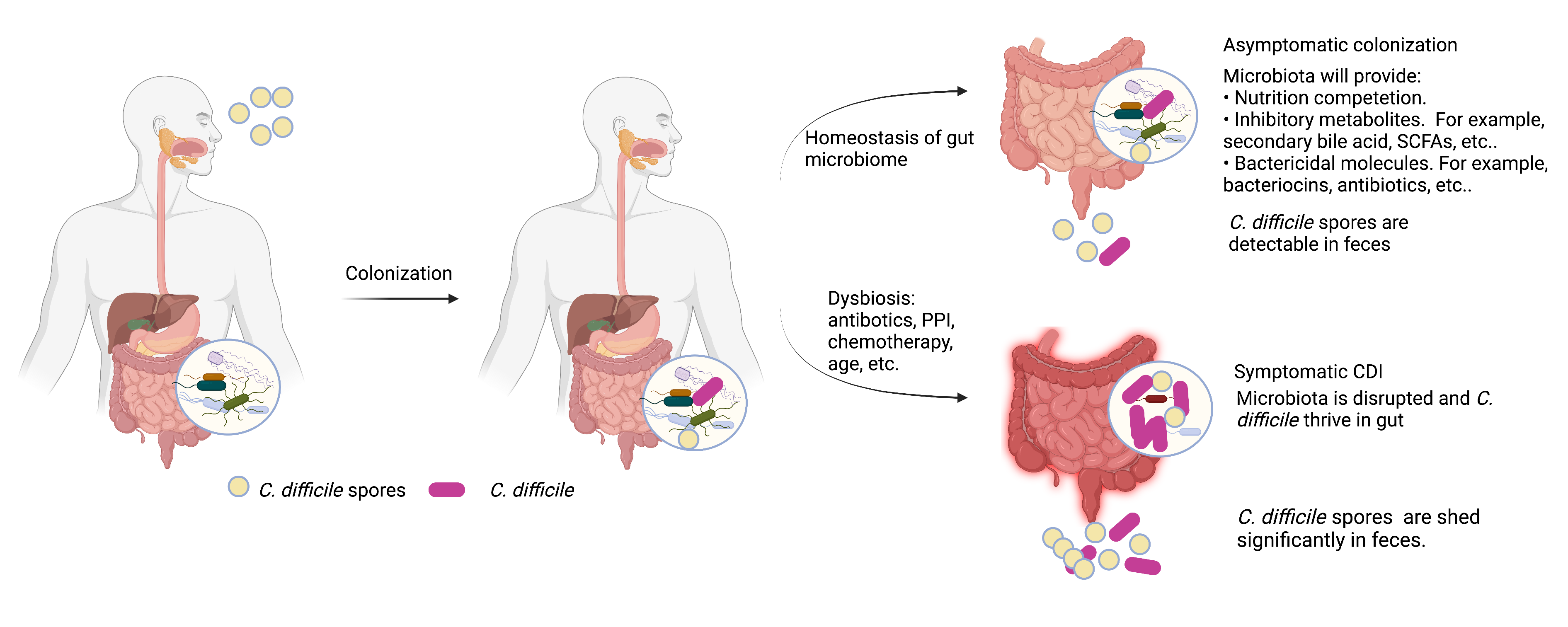

Supplement: Supplemental Material [file KGMI_A_2052698_SM1940.png]
